# Prognostic relevance of sarcopenia, geriatric, and nutritional assessments in older patients with diffuse large B-cell lymphoma: results of a multicentric prospective cohort study

**DOI:** 10.1007/s00277-023-05200-x

**Published:** 2023-04-14

**Authors:** Juliette Pénichoux, Hélène Lanic, Caroline Thill, Anne-Lise Ménard, Vincent Camus, Aspasia Stamatoullas, Emilie Lemasle, Stéphane Leprêtre, Pascal Lenain, Nathalie Contentin, Jerôme Kraut-Tauzia, Christophe Fruchart, Leila Kammoun, Gandhi Damaj, Agathe Farge, Caroline Delette, Romain Modzelewski, Sandrine Vaudaux, Louis-Ferdinand Pépin, Hervé Tilly, Fabrice Jardin

**Affiliations:** 1 Department of Clinical Hematology, Centre Henri Becquerel, 1 Rue d’Amiens, 76038 Rouen, France; 2 Department of Statistics, Rouen University Hospital, Rouen, France; 3 INSERM U1245 Unit, Team “Genetic and Biomarkers in Lymphoma and Solid Tumors”, Rouen University, Centre Henri Becquerel, Rouen, France; 4 Wilson Imaging Center, Strasbourg, France; 5 Department of Clinical Hematology, CHD, Dunkerque, France; 6 Department of Oncology-Hematology, Eure-Seine Hospital Center, Evreux, France; 7 Institute of Hematology, Caen University Hospital, Caen, France; 8 Department of Clinical Hematology, Amiens University Hospital, Amiens, France; 9 Department of Nuclear Medicine, Centre Henri Becquerel, Rouen, France; 10 Clinical Research Unit, Henri Becquerel Cancer Center, Rouen, France

**Keywords:** Sarcopenia, Diffuse large B-cell lymphoma, Nutritional support, Geriatric evaluation

## Abstract

This prospective study aimed to investigate the prognostic effect of sarcopenia, geriatric, and nutritional status in older patients with diffuse large B-cell lymphoma (DLBCL). Ninety-five patients with DLBCL older than 70 years who were treated with immunochemotherapy were included. The lumbar L3 skeletal muscle index (L3-SMI) was measured by computed tomography at baseline, and sarcopenia was defined as low L3-SMI. Geriatric assessment included G8 score, CIRS-G scale, Timed Up and Go test, and instrumental activity of daily living. Nutritional status was assessed using the Mini Nutritional Assessment and the body mass index, and several scores used in the literature incorporating nutritional and inflammatory biomarkers, namely the Nutritional and inflammatory status (NIS), Geriatric Nutritional Risk Index, Prognostic Nutritional Index, and Glasgow Prognostic Score.

Fifty-three patients were considered sarcopenic. Sarcopenic patients displayed higher levels of inflammation markers and lower levels of prealbumin than non-sarcopenic patients. Sarcopenia was associated with NIS, but was not associated with severe adverse events and treatment disruptions. They were, however, more frequent among patients with elevated NIS. Sarcopenia did not appear in this study as a prognostic factor for progression-free survival (PFS) or overall survival (OS). However, NIS emerged as predictive of the outcome with a 2-year PFS rate of 88% in the NIS ≤ 1 group and 49% in the NIS > 1 group and a significant effect in a multivariate analysis for both PFS (*p* = 0.049) and OS (HR = 9.61, CI 95% = [1.03–89.66], *p* = 0.04). Sarcopenia was not associated with adverse outcomes, but was related to NIS, which appeared to be an independent prognostic factor.

**Supplementary Information:**

The online version contains supplementary material available at 10.1007/s00277-023-05200-x.

## Background

Diffuse large B-cell lymphoma (DLBCL) is the most common non-Hodgkin lymphoma, and its incidence increases with age, with a median age at diagnosis of 70 years [1]. Patients with DLBCL above 60 years of age are typically treated with cyclophosphamide, doxorubicin, vincristine, and prednisone plus rituximab (R-CHOP). More recently, the POLARIX trial provided an alternative named pola-R-CHP, in which vincristine is replaced with polatuzumab vedotin [2]. Due to their frequent comorbidities, standard chemotherapy regimens in older patients are often associated with non-manageable levels of toxicity which may compromise the optimal course of treatment [3]. A proper selection of older patients eligible for aggressive chemotherapy is therefore necessary. Reduced intensity R-miniCHOP [4], in which cyclophosphamide, doxorubicin, and vincristine are administered at lower doses, is considered the gold standard treatment in patients older than 80 or considered unfit [5].

Several studies have searched prognostic factors in older patients with DLBCL. A comprehensive geriatric assessment using age in conjunction with instruments evaluating the activity of daily living, the instrumental activity of daily living (IADL), and comorbidities using the Cumulative Illness Rating Score for Geriatrics (CIRS-G) was used to define categories of “fit,” “unfit,” and “frail” [6–9] with significant prognostic impact [6, 7, 9–15].

Numerous studies have highlighted the impact of nutritional status on the prognosis of DLBCL, particularly in older patients [4, 16–22]. Nutritional and inflammatory status (NIS) [23], a score based on albumin, prealbumin, and two markers of inflammation, namely, C-reactive protein (CRP) and alpha-1 acid glycoprotein, was associated with toxicity following chemotherapy in patients with cancer [24] and with overall survival (OS) in metastatic breast cancer [25]. To our knowledge, it has not been evaluated in lymphoma.

The relationship between body composition, specifically the proportion of lean and fat tissues, and cancer outcomes has been of recent interest. Adipopenia was an adverse prognostic factor in older patients with DLBCL in one study [26]. Sarcopenia, defined by the depletion of skeletal muscle, has been recognized as an unfavorable prognostic factor and predictor of chemotherapy toxicity in older patients with solid tumors [27–30]. In patients with DLBCL, several studies showed that sarcopenic patients had poor outcomes in terms of survival and progression-free survival (PFS) [31–35] as well as treatment-related mortality and treatment discontinuation [33, 36, 37]. However, other studies did not retain sarcopenia as an independent prognostic factor [38–42]. To our knowledge, three studies have focused on older patients. Two retrospective studies by Lanic et al. [31] and Camus et al. [26] showed that sarcopenia was an independent adverse prognostic factor in a population of patients over 70 years old treated with rituximab and chemotherapy. Conversely, in a subgroup analysis of older patients, Chu et al. [41] suggested an improved OS in patients with sarcopenia compared to non-sarcopenic patients. Therefore, the prognostic impact of sarcopenia remains controversial in older patients with DLBCL.

This multicentric cohort study aims to prospectively evaluate the prognostic impact of sarcopenia and geriatric and nutritional status, in patients with DLBCL over 70 years old treated with chemotherapy and rituximab and to better characterize sarcopenic patients with respect to other known prognostic factors.

## Methods

### Study design and patients

All patients diagnosed with DLBCL in one of the participating centers between January 2012 and April 2014 who fulfilled the inclusion criteria and agreed to sign an informed consent form were consecutively enrolled in this multicentric prospective study. The participating centers were 8 French hematology departments.

The inclusion criteria were histologically proven DLBCL or grade III follicular lymphoma, age over 70, and treatment with R-CHOP (rituximab 375mg/m^2^, cyclophosphamide 750 mg/m^2^, vincristine 1.4 mg/m^2^, doxorubicin 50 mg/m^2^, prednisone 40 mg/m^2^ on days 1 to 5) or R-miniCHOP (rituximab 375mg/m^2^, cyclophosphamide 400 mg/m^2^, vincristine 1 mg/m^2^, doxorubicin 25 mg/m^2^, prednisone 40 mg/m^2^ on days 1 to 5). The exclusion criteria were low-grade transformed lymphoma, the impossibility of performing a computed tomography (CT)-scan, positive serology for human immunodeficiency virus, hepatitis C virus or hepatitis B virus, and the impossibility of using anthracyclines.

Patients underwent a clinical evaluation every 3 months and an assessment by a CT scan at 3, 6, 12, 18, and 24 months after inclusion or in case of treatment disruption. Patients were followed until May 2016. The trial was registered (ClinicalTrials.gov identifier: NCT01715961), approved by the “Comité de protection des personnes,” and performed according to the Helsinki Rules.

### Geriatric assessment

The geriatric screening score G8 was used [43], ranging from 0 (heavily impaired) to 17 (not at all impaired), with scores below 14 being considered subnormal. Functional assessment was made using the Timed Up and Go test [44] and Barberger-Gateau’s four-item IADL scale [45], which ranges from 0 (dependent) to 4 (independent) and includes telephone use, the correct use of medicines, transportation, and management of finances. The handgrip strength (in kilograms) [46] was measured using a hand dynamometer with each hand. Comorbidities were assessed using the CIRS-G scale [47]. Patients with CIRS-G > 7 were considered to have pronounced comorbidities [48].

### Nutritional assessment

Nutritional status was assessed clinically through the body mass index (BMI) and the Mini Nutritional Assessment (MNA) [49] on a 0 to 30 scale, with scores above 24 being normal, below 17 corresponding to malnutrition, and between 17 and 24 to patients at risk of malnutrition. Additional assessment of nutritional status through biological parameters was obtained through albumin and the NIS, Prognostic Nutritional Index (PNI) [50], Geriatric Nutritional Risk Index (GNRI) [51], and Glasgow Prognostic Score (GPS) [22]. The NIS was calculated as previously described [23] as the ratio (CRP (mg/L) × alpha-1 acid glycoprotein (g/L))/(albumin(g/L) × prealbumin (g/L)). The PNI was calculated as albumin (g/L) + 5 × lymphocyte count (10^9^/L). A PNI lower than 45 was considered subnormal [17, 52]. The GNRI was calculated from body weight and albumin as 14.89 × albumin (g/dL) + 41.7 × (body weight /ideal body weight). Ideal body weight was defined as 22 × [height (m)]^2^. The body weight/ideal body weight was defined as 1 when the patient’s body weight exceeded the ideal body weight. As in previous studies, patients were categorized into four groups according to the GNRI value: 0, no risk (> 98); 1, mild risk (92–98); 2, moderate risk (82 to 92); and 3, severe risk (< 82) [51]. As in previous reports [22], patients with CRP levels below 10 mg/L and albumin level above 35 g/L were given a GPS score of 0. Patients with either CRP > 10 mg/L or albumin level < 35 g/L were allocated a score of 1, while patients with both CRP > 10 mg/L and albumin level < 35 g/L received a score of 2.

### Sarcopenia assessment and CT scan imaging

As reported in other studies [53, 54], muscle mass and fat tissues were measured by analyzing CT images obtained prior to treatment. A lumbar vertebral landmark L3 was used because this region’s skeletal muscle and fat tissue correspond to the whole-body tissue quantities [55]. The surfaces of the different tissues were selected according to the CT Hounsfield unit, ranging from −29 to 150 for skeletal muscles and −190 to −30 and −150 to −50 for subcutaneous and visceral adipose tissue, respectively. A Hounsfield unit-based analysis of the images was performed using dedicated software, LITIS EA 4108, which was developed in our laboratory, to segment fat and lean tissue and quantify the cross-sectional area (cm^2^) of each tissue type by summing the given tissue’s pixels and multiplying the sum by the pixel surface area. The tissue boundaries were manually corrected as necessary. Two adjacent images at the third lumbar level were used to measure each tissue’s surface area and averaged. The values obtained were normalized for stature to calculate the lumbar L3 skeletal muscle index (L3-SMI) and the lumbar L3 visceral adipose and subcutaneous tissue indexes (L3-VAI and L3-SAI) (cm^2^/m^2^).

### Definition of sarcopenia and adipopenia

As previously reported [31], women with an L3-SMI below 38.9 cm^2^/m^2^ and men with an L3-SMI below 55.8 cm^2^/m^2^ were considered sarcopenic. As described in Camus et al. [26], L3-VAI was considered low (visceral adipopenia) when below 50.4 cm^2^/m^2^ in men and below 43.5 cm^2^/m^2^ in women. L3-SAI was considered low (subcutaneous adipopenia) when below 47.4 cm^2^/m^2^ in men and below 76.3 cm^2^/m^2^ in women.

### Treatment toxicities

The Common Terminology Criteria for Adverse Events (CTCAE) version 4 was prospectively applied to collect and grade all toxicities after each cycle. All toxicities were included except for cytopenia without complications. Febrile neutropenia was taken into account. Adverse events’ grades 3–5 were considered severe adverse events.

### Statistical analysis

OS was calculated from the date of enrollment to death from any cause. PFS was calculated from enrollment until disease progression, relapse, or death. Patients without PFS or OS events were censored at the last date of follow-up.

Survival curves were estimated using the Kaplan-Meier method and compared using the likelihood-ratio test. Multivariate analysis was performed with a Cox proportional hazards regression model with backward stepwise selection, integrating the International Prognostic Index (IPI), bulky disease, lymphopenia, and hypoalbuminemia as the main known prognostic factors, and sarcopenia as main variable of interest. Hazard ratios (HR) and corresponding 95% confidence intervals (CI) are presented. To address the missing data, a sensitivity analysis was conducted using multiple imputation by chained equations.

Comparisons between sarcopenic and non-sarcopenic patients for categorical data were performed using Pearson chi-square test or Fisher’s exact test when necessary. For continuous data, Student’s *t*-test and Mann–Whitney *U* test were used for normally and non-normally distributed data, respectively. All tests were two-sided, and *p*-values lower than 0.05 were regarded as statistically significant. Analyses were performed with R software.

## Results

### Patient characteristics

Ninety-seven patients were enrolled in the study and followed until May 2016. Two patients were excluded from the study because of withdrawal of consent and inclusion by mistake of a patient with exclusion criteria. Nine patients did not have L3-SMI measurements available, because of ascites, tumoral infiltration of the muscle, or morbid obesity.

Baseline characteristics are summarized in Table 1. The mean age was 78.4 years and ranged between 70 and 92. Men and women were equally represented (49 vs. 51%). Most patients had an advanced-stage disease (68%), with more than 1 extranodal sites in 35 patients (37%), B symptoms in 30 patients (32%), and bulky disease in 35 patients (37%). The majority had a good performance status (Eastern Cooperative Oncology Group performance status (ECOG-PS) <2) (62%) but had at least 3 concomitant drugs (87%). Fifty-four patients (57%) received R-CHOP, and 40 (42%) received an R-miniCHOP regimen.

**Table 1:** Patient clinical and disease characteristics at diagnosis and comparison between patients with or without sarcopenia

	Total (*n*=95)	Non-sarcopenic (*n*=33)	Sarcopenic (*n*=53)	*p*-value
Age (mean [sd])	78.4 [5.3]	78.4 [4.9]	78.7 [5.3]	0.78
Sex				**<0.0001**
Male	47 (49%)	7 (21%)	35 (66%)	
Female	48 (51%)	26 (79%)	18 (34%)	
Number of treatments	(NA = 8)	(NA = 3)	(NA = 5)	0.14
< 3	11 (13%)	1 (3%)	8 (17%)	
≥ 3	76 (87%)	29 (97%)	40 (83%)	
Stage				0.18
I–II	30 (32%)	14 (42%)	15 (28%)	
III–IV	65 (68%)	19 (58%)	38 (72%)	
ECOG-PS				0.16
<2	59 (62%)	25 (76%)	32 (60%)	
≥ 2	36 (38%)	8 (24%)	21 (40%)	
IPI	(NA = 1))	(NA = 1)		0.09
0–2	56 (60%)	24 (75%)	30 (57%)	
3–4	38 (40%)	8 (25%)	23 (43%)	
Nb of extranodal sites				**0.04**
≤ 1	60 (63%)	26 (79%)	30 (57%)	
> 1	35 (37%)	7 (21%)	23 (43%)	
Treatment	(NA = 1)	(NA = 1)		0.72
R-CHOP	54 (57%)	18 (55%)	31 (58%)	
R-miniCHOP	40 (42%)	15 (45%)	22 (42%)	
B-symptoms				0.86
No	65 (68%)	23 (70%)	36 (68%)	
Yes	30 (32%)	10 (30%)	17 (32%)	
Bulky disease (>10 cm)				0.24
No	60 (63%)	24 (73%)	32 (60%)	
Yes	35 (37%)	9 (27%)	21 (39%)	

*Sd*, standard deviation; *IPI*, International Prognostic Index; *ECOG-PS*, Eastern Cooperative Oncology Group performance status; *R-CHOP*, rituximab, cyclophosphamide, doxorubicin, oncovin; *NA*, not available. Bold values denote statistical significance at the *p *< 0.05 level

The biological features at baseline are summarized in Table 2. The median lymphocyte count was 0.9 G/L. Most patients had an increased lactate dehydrogenase (LDH) level (71%). The mean nutritional biological markers were below or at the lower limit of normal ranges with a mean albumin level of 3.4 g/dL and a mean transthyretin level of 0.2 g/L. Inflammatory markers were above normal, with a mean CRP of 29.3 mg/L and a mean alpha-1 acid glycoprotein of 1.4 g/L.

**Table 2:** Biological features at baseline and comparison between patients with or without sarcopenia

	Total (*n*=95)	Non-sarcopenic (*n*=33)	Sarcopenic (*n*=53)	*p*-value
LDH				0.21
Normal	28 (29%)	13 (39%)	14 (26%)	
Increased	67 (71%)	20 (61%)	39 (74%)	
Lymphocyte count				0.19
< 0.9 G/L	45 (47%)	12 (36%)	27 (51%)	
> 0.9 G/L	50 (53%)	21 (64%)	26 (49%)	
Albumin (g/dL) (mean [sd])	3.4 [0.6] (NA = 4)	3.5 [0.7] (NA = 1)	3.4 [0.6] (NA = 3)	0.41
Prealbumin (g/L) (mean [sd])	0.20 [0.1] (NA = 9)	0.22 [0.1] (NA = 3)	0.18 [0.1] (NA = 6)	**0.01**
Alpha-1 acid glycoprotein (g/L) (mean [sd])	1.4 [0.5] (NA=10)	1.2 [0.4]	1.4 [0.5] (NA = 8)	**0.03**
CRP (mg/L) (mean [sd])	29.3 [38.6] (NA = 5)	15.4 [23.8] (NA = 1)	33.7 [41] (NA = 3)	**0.02**

*Sd*, standard deviation; *LDH*, lactate dehydrogenase; *CRP*, C-reactive protein; *NA*, not available. Bold values denote statistical significance at the *p* < 0.05 level

The results for anthropometric, nutritional, and geriatric parameters are shown in Table 3. The mean BMI was 26.2 kg/m^2^, with half of the patients with a BMI below 25, 15 (16%) obese patients, and a subnormal MNA score in 46 patients (59%). Thirty-two patients (39%) had a low L3-SAI, and 38 (44%) had a low L3-VAI. NIS was above 1 in 63% of the patients. Most patients had a low PNI (71%) and a GPS above 0 (68%), and 27% were not at risk according to the GNRI. G8 score was subnormal in most patients (73%). The IADL score was below 4 in 22 patients (23%), and the Timed Up and Go test was above 20 s in 26% of the patients. Twenty-four patients (25%) were considered to have pronounced comorbidities. Among the 86 patients with available data, the mean L3-SMI was 44.9, with 53 patients (62%) considered sarcopenic.

**Table 3:** Anthropometric, nutritional, and geriatric features and comparison between patients with or without sarcopenia

	Total (*n*=95)	Non-sarcopenic (*n*=33)	Sarcopenic (*n*=53)	*p*-value
BMI (kg/m^2^) (mean[sd])	26.2 [5.6] (NA = 1)	28.4 [5.5] (NA = 1)	24.8 [5.1]	**0.003**
MNA score	(NA = 17)	(NA = 7)	(NA = 8)	0.64
<17	4 (5%)	0	3 (7%)	
17–24	42 (54%)	15 (58%)	25 (56%)	
>24	32 (41%)	11 (42%)	17 (38%)	
L3-SMI (mean ([sd])	44.9 [9.7] (NA = 9)	49.3 [11,0]	42.2 [7.7]	
Low L3-SAI*	(NA = 13)	(NA = 2)	(NA = 2)	0.33
No	50 (61%)	21 (68%)	29 (57%)	
Yes	32 (39%)	10 (32%)	22 (43%)	
Low L3-VAI*	(NA = 9)			0.11
No	48 (56%)	22 (67%)	26 (49%)	
Yes	38 (44%)	11 (33%)	27 (51%)	
NIS score	(NA = 23)	(NA = 5)	(NA = 15)	**0.002**
≤1	27 (37%)	18 (64%)	9 (24%)	
>1	45 (63%)	10 (36%)	29 (76%)	
Low PNI	(NA = 4)	(NA = 1)	(NA = 3)	0.90
No	26 (29%)	10 (31%)	15 (30%)	
Yes	65 (71%)	22 (69%)	35 (70%)	
GNRI	(NA = 5)	(NA = 2)	(NA = 3)	0.50
0- No risk	24 (27%)	12 (39%)	12 (24%)	
1- Low risk	26 (29%)	8 (26%)	15 (30%)	
2- Moderate risk	25 (28%)	6 (19%)	15 (30%)	
3- Severe risk	15 (17%)	5 (16%)	8 (16%)	
GPS	(NA = 9)	(NA = 2)	(NA = 6)	**0.01**
0	27 (31%)	17 (55%)	10 (21%)	
1	27 (31%)	6 (19%)	18 (38%)	
2	32 (37%)	8 (26%)	19 (40%)	
G8 score	(NA = 1)		(NA = 1)	0.30
≤14	69 (73%)	22 (67%)	40 (77%)	
>14	25 (27%)	11 (33%)	12 (23%)	
IADL score (/4)				0.13
<4	22 (23%)	11 (33%)	10 (19%)	
4	73 (77%)	22 (67%)	43 (81%)	
Timed Up and Go test	(NA = 21)	(NA = 5)	(NA = 13)	
Inability to perform	4 (5%)	1 (4%)	3 (8%)	
Time ≤ 20 s	51 (69%)	20 (71%)	27 (68%)	0.92
Time > 20 s	19 (26%)	7 (25%)	10 (25%)	
CIRS-G score (mean ([sd]))	5.63 [3.80] (NA = 2)	5.75 [3.64]	5.69 [4.13] (NA = 2)	0.90
≤ 7	69 (74%)	24 (73%)	37 (73%)	
> 7	24 (25%)	9 (27%)	14 (27%)	

*BMI*, body mass index; *MNA*, Mini Nutritional Assessment; *L3-SMI*, lumbar L3 skeletal muscle index; *L3-VAI*, lumbar L3 visceral adipose tissue index; *L3-SAI*, lumbar L3 subcutaneous adipose tissue index; *NIS*, nutritional and inflammatory status; *PNI*, Prognostic Nutritional Index; *GNRI*, Geriatric Nutritional Risk Index; *GPS*, Glasgow Prognostic Score; *IADL*, instrumental activities of daily living; *CIRS-G*, Cumulative Illness Rating Scale-Geriatric; *NA*, not available. Bold values denote statistical significance at the *p* < 0.05 level

*Low L3-SAI: male L3-SAI < 47.4 cm^2^/m^2^, female L3-SAI < 76.3 cm^2^/m^2^

Low L3-VAI: male L3-VAI < 50.4 cm^2^/m^2^, female L3-VAI < 43.5 cm^2^/m^2^

### Association between sarcopenia and other factors

Sarcopenia was more frequent among men than women (*p* < 0.0001), and among patients with extranodal sites (*p* = 0.04). No significant differences were found between sarcopenic and non-sarcopenic patients for age, performance status, polypharmacy, stage of the disease, presence of B-symptoms, or bulky disease (Table 1). The proportions of patients treated with R-CHOP or R-miniCHOP were similar in the two groups.

Sarcopenic patients displayed similar levels of LDH and albumin when compared to non-sarcopenic patients. However, sarcopenia was associated with a lower level of prealbumin (*p* = 0.01) and higher levels of alpha-1 acid glycoprotein (*p* = 0.03) and CRP (*p* = 0.02) (Table 2). Sarcopenic patients had a lower BMI (24.8 kg/m^2^ vs. 28.4 kg/m^2^, *p* = 0.003). The NIS score was below 1 among most non-sarcopenic patients (64%), while it was increased in a majority of sarcopenic patients (76%, *p* = 0.002). Similarly, a majority of non-sarcopenic patients had a GPS of 0 (55%), compared to only 21% in the sarcopenic group (*p* = 0.01). Sarcopenic and non-sarcopenic patients did not differ in terms of MNA score, the proportion with low L3-VAI or L3-SAI, PNI, GNRI, G8 score, IADL, or Timed Up and Go test. The handgrip strength tests in men and women did not differ between sarcopenic and non-sarcopenic patients (Table 4).

**Table 4:** Handgrip strength test among men and women and comparison between patients with or without sarcopenia

	Men (*n*=42)			Women (*n* = 44)		
	Non-sarcopenic (*n* = 7)	Sarcopenic (*n* = 35)	*p*-value	Non-sarcopenic (*n* = 26)	Sarcopenic (*n* = 18)	*p*-value
Handgrip Strength						
Left hand (mean [sd])	36.9 [25.8]	37.5 [17.6]	0.53	20.0 [8.6] (NA = 1)	20.5 [12.5]	0.58
Right hand (mean [sd])	35.9 [22.8]	38.8 [17.7] (NA = 1)	0.53	21.2 [8.5]	22.6 [15.7]	0.34

*Sd*, standard deviation; *NA*, not available

### Treatment toxicity

During the whole duration of treatment, 67 patients (70%) experienced no severe adverse events (aside cytopenia without complication), 15 (16%) patients experienced one severe adverse event, and 13 (14%) experienced more than one severe adverse event.

Table 5 summarizes the number of cycles of treatment administered and the occurrence of severe adverse events during the first cycle of treatment. Most patients received between 6 and 8 cycles (78%), without any significant difference between sarcopenic and non-sarcopenic patients (*p* = 0.62). Severe adverse events (grades 3–5) occurred in 14 patients (15%) during the first cycle, with similar frequencies among sarcopenic and non-sarcopenic patients (*p* = 0.87). Patients with NIS above 1 were more likely to discontinue treatment, with 24% receiving less than 6 cycles (*p* = 0.005), while none of the patients with NIS below 1 discontinued before the 6th cycle. Toxicity was also more frequent in this group (*p* = 0.02), with 16% experiencing a severe adverse event during the first cycle.

**Table 5:** Number of cycles administered and toxicity during the first cycle of chemotherapy

	Total	Sarcopenia			NIS		
	(*n*=95)	Non-sarcopenic (*n* = 33)	Sarcopenic (*n* = 53)	*p*-value	NIS ≤1 (*n* = 27)	NIS>1 (*n* = 45)	*p*-value
Number of cycles administered				0.62			**0.005**
<6	21 (22%)	6 (18%)	12 (23%)		0 (0%)	11 (24%)	
6–8	74 (78%)	27 (64%)	41 (77%)		27 (100%)	34 (76%)	
Adverse events’ grades 3–5 during the first cycle of treatment*				0.87			**0.02**
0	81 (85%)	29 (88%)	45 (85%)		26 (96%)	38 (84%)	
1	12 (13%)	3 (9%)	7 (13%)		0	7 (16%)	
2	2 (2%)	1 (3%)	1 (2%)		1 (4%)	0	

*Does not include cytopenias without complications. Bold values denote statistical significance at the *p* < 0.05 level

Supplementary Table 1 compares the results according to treatment with R-CHOP or R-miniCHOP. Severe adverse events during cycle 1 were more frequent among patients treated with R-CHOP (22%) than R-miniCHOP (6%, *p* = 0.01), but there was no significant difference in treatment discontinuations. R-CHOP proved particularly toxic among patients with NIS > 1, with 28% experiencing a severe adverse event at cycle 1 vs. 0% for R-miniCHOP (*p* = 0.01).

### Progression-free survival and overall survival

**Fig. 1:**
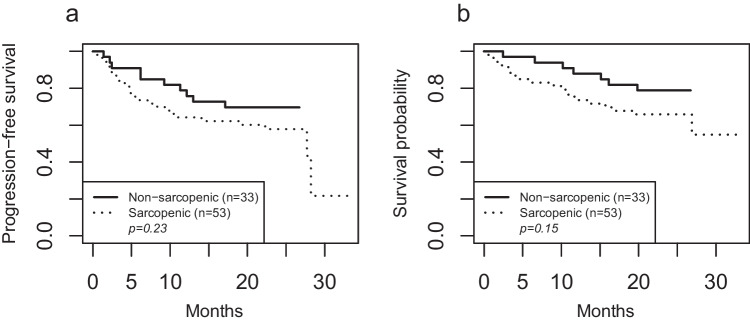
Progression-free survival (**a**) and overall survival (**b**) in patients with and without sarcopenia

The median follow-up was 22.7 months. Thirty-one patients progressed and 30 patients died during the study. There was no significant difference in PFS between sarcopenic and non-sarcopenic patients (Fig. 1a, *p* = 0.23). The 2-year PFS rate was 70% in the non-sarcopenic group and 58% in the sarcopenic group. Sarcopenia did not show any association with OS (Fig. 1b, *p* = 0.15). The 2-year OS rate was 79% in the non-sarcopenic group and 66% in sarcopenic patients.

**Fig. 2:**
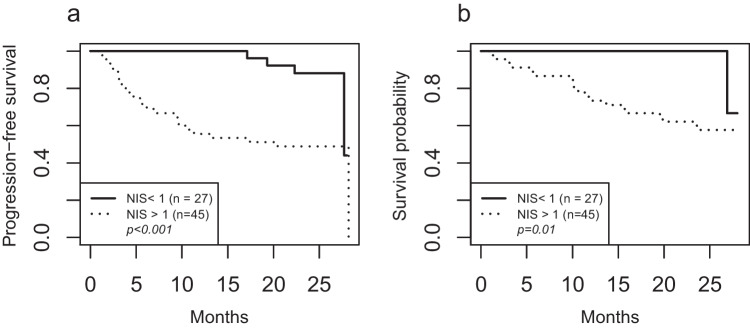
Progression-free survival (**a**) and overall survival (**b**) in patients with NIS < 1 and NIS > 1

There was a significant difference in PFS between patients with NIS below or above 1 (Fig. 2a, HR=5.08, 95% CI = [1.75–14.78], *p* < 0.001). The 2-year PFS rate was 88% in the NIS ≤ 1 group and 49% in the NIS > 1 group. There was a significant difference in OS between patients with NIS below or above 1 (Fig. 2b, HR = 13.74, 95% CI = [1.83–103.1], *p* = 0.01). No patient in the NIS ≤ 1 group died before 2 years. The 2-year OS rate was 58% in the NIS > 1 group.

The outcomes did not differ between patients receiving R-CHOP and patients receiving R-miniCHOP in the whole cohort (Table 6), or in the subgroups of either sarcopenic or non-sarcopenic patients (Supplementary Fig. 1), or among patients with NIS>1 (Supplementary Fig. 2).

**Table 6:** Multivariate analysis for progression-free survival and overall survival

	PFS				OS			
	Univariate		Multivariate		Univariate		Multivariate	
	HR [95% CI]	*p*	HR [95% CI]	*p*	HR [95% CI]	*p*	HR [95% CI]	*p*
Sarcopenia	1.56 [0.74–3.30]	0.23	0.87 [0.31–2.46]	0.79	1.84 [0.77–4.41]	0.15	1.17 [0.31–4.41]	0.82
Gender, female	0.51 [0.27–0.98]	**0.04**	0.22 [0.08–0.61]	**0.004**	0.59 [0.28–1.23]	0.15	0.34 [0.11–1.02]	0.053
Age > 80 yr	1.41 [0.75–2.66]	0.29	-		1.28 [0.62–2.67]	0.50	-	
Stage III–IV	2.69 [1.19–6.10]	**0.01**	-		2.02 [0.82–4.94]	0.10	-	
> 1 extranodal site	1.96 [1.05–3.65]	**0.03**	-		2.32 [1.13–4.76]	**0.02**	-	
B-symptoms	2.14 [1.14–3.99]	**0.02**	-		1.92 [0.93–3.97]	0.08	-	
Bulky disease (> 10 cm)	2.03 [1.09–3.78]	**0.03**	2.82 [1.09–7.25]	**0.03**	1.96 [0.96–4.02]	0.06	2.00 [0.66–6.08]	0.22
ECOG-PS ≥ 2	1.43 [0.76–2.70]	0.27	-		1.84 [0.90–3.77]	0.10	-	
IPI ≥ 3	2.10 [1.13–3.93]	**0.02**	0.88 [0.33–2.39]	0.81	2.22 [1.07–4.58]	**0.03**	1.08 [0.33–3.52]	0.89
BMI (kg/m2)		0.51	-			0.36	-	
< 25	1		-		1		-	
Overweight ([25 ; 30])	0.95 [0.46–1.93]		-		0.74 [0.31–1.78]		-	
Obese (> 30)	1.59 [0.69–3.63]		-		1.60 [0.65–3.93]		-	
Low L3-VAI*	0.44 [0.21–0.93]	**0.02**	0.38 [0.15–1.01]	0.052	0.49 [0.21–1.14]	0.09	0.30 [0.09–1.00]	**0.050**
Low L3-SAI*	0.70 [0.33–1.49]	0.35	-	-	0.70 [0.30–1.64]	0.41	-	-
Lymphopenia	2.58 [1.34–4.99]	**0.005**	2.12 [0.83–5.42]	0.12	3.26 [1.49–7.16]	**0.003**	2.86 [0.86–9.55]	0.09
Hypoalbuminemia	11.86 [1.61–87]	**0.01**	4.64 [0.54–39.57]	0.16	7.64 [1.03–56.46]	**0.01**	1.49 [0.15–15.26]	0.74
NIS > 1	5.08 [1.75–14.78]	**< 0.001**	3.48 [1.00–12.08]	**0.049**	13.74 [1.83–103.1]	**0.01**	9.61 [1.03–89.66]	**0.04**
LDH>UNL	2.45 [1.08–5.54]	**0.02**	-		2.40 [0.92–6.27]	**0.05**	-	
Timed Up and Go test > 20s	1.80 [0.82–3.94]	0.15	-		1.85 [0.77–4.48]	0.18	-	
Hand grip test (right)	1.00 [0.98–1.02]	0.77	-		1.00 [0.98–1.02]	0.76	-	
G8 < 14	0.90 [0.45–1.82]	0.78	-		0.97 [0.43–2.18]	0.94	-	
MNA		0.87	-			0.38	-	
<17	0.61 [0.08–4.63]		-		1.40 [0.17–11.45]		-	
17–24	0.96 [0.48–1.93]		-		1.86 [0.75–4.56]		-	
>24	1		-		1		-	
IADL score (/4) < 4	1.50 [0.74–3.02]	0.27	-		2.33 [1.10–4.95]	**0.03**	-	
CIRS-G score >7	1.38 [0.69–2.77]	0.37	-		1.31 [0.60–2.85]	0.50	-	
PNI < 45	2.20 [0.97–5.02]	**0.04**	-		2.89 [1.001–8.33]	**0.03**	-	
GNRI, categories	1.57 [1.18–2.08]	**< 0.001**	-		1.58 [1.14–2.19]	**0.003**	-	
GPS		**< 0.001**	-			**< 0.001**	-	
0	1				1			
1	4.29 [1.41–13.07]				5.31 [1.15–24.60]			
2	6.54 [2.19–19.55]				9.51 [2.18–41.42]			
Treatment with R-miniCHOP	0.79 [0.41–1.54]	0.50	-		0.77 [0.36–1.63]	0.49	-	

*PFS*, progression-free survival; *OS*, overall survival; *HR*, hazard ratio; *CI*, confidence interval; *ECOG-PS*, Eastern Cooperative Oncology Group performance status; *IPI*, International Prognostic Index; *BMI*, body mass index; *L3-VAI*, lumbar L3 visceral adipose tissue index; *L3-SAI*, lumbar L3 subcutaneous adipose tissue index; *NIS*, nutritional and inflammatory status; LDH lactate dehydrogenase; UNL, upper normal limit; MNA mini nutritional assessment; IADL, instrumental activities of daily living; *CIRS-G*, Cumulative Illness Rating Scale-Geriatric; *PNI*, prognostic nutritional index; *GNRI*, Geriatric Nutritional Risk Index; *GPS*, Glasgow Prognostic Score. Bold values denote statistical significance at the *p* < 0.05 level

*Low L3-SAI: male L3-SAI < 47.4 cm^2^/m^2^, female L3-SAI < 76.3 cm^2^/m^2^

Low L3-VAI: male L3-VAI < 50.4 cm^2^/m^2^, female L3-VAI < 43.5 cm^2^/m^2^

The results of the univariate and multivariate analyses for PFS and OS are shown in Table 6, and 2-year PFS and OS are provided in Supplementary Table 2. In addition to NIS, other factors identified as prognostic factors for PFS in univariate analysis were male sex, stage III/IV, more than 1 extranodal site, B-symptoms, bulky disease, IPI≥3, lymphopenia, hypoalbuminemia, increased LDH, and the various nutritional and inflammation indices, namely PNI, GNRI, and GPS. Visceral adipopenia (low L3-VAI) was associated with a longer PFS (*p* = 0.02). The factors associated with lower OS in addition to NIS in univariate analysis were more than 1 extranodal site, lymphopenia, hypoalbuminemia, increased LDH, IADL score, PNI, GNRI, and GPS. Conversely, age, ECOG-PS, a low L3-SAI, Timed Up and Go test, G8 score, and IADL were not significantly associated with either PFS or OS, and gender, stage III/IV, B-symptoms, bulky disease, and BMI were associated with PFS but not with OS. Factors that retained prognostic value in the multivariate analysis were male sex (*p* = 0.004), NIS > 1 (*p* = 0.049) and bulky disease (*p* = 0.03) for PFS, and NIS >1 (*p* = 0.04) for OS. A sensitivity analysis with multiple imputation for missing data shows very similar results (Supplementary Table 3).

To further compare the prognostic impact of GPS and NIS, a model incorporating these two scores showed a significant effect of NIS, while GPS became non-significant (data not shown).

## Discussion

In the present study, sarcopenia, assessed by a CT scan through the lumbar L3 muscle index, was not significantly associated with PFS or OS in patients with DLBCL over 70 years old. The prognostic impact of sarcopenia in patients with DLBCL has not been consistently observed in all studies. Several studies showed an independent prognostic effect [26, 31–34], including two studies focusing on older patients [26, 31]. However, other studies did not corroborate this effect [38, 39, 41, 42], including an analysis focusing on older patients [41], or reported a prognostic impact limited to male patients [40]. This study is, to our knowledge, the first to prospectively analyze sarcopenia as well as a large panel of geriatric and nutritional parameters in older patients with DLBCL. Our modest sample size may lead to a lack of power that could explain this negative result for sarcopenia, and does not enable us to perform a subgroup analysis by gender to assess the hypothesis of an adverse effect of sarcopenia limited to male patients, as reported by Nakamura et al. Sarcopenia was, however, more frequent among men. Men had less favorable outcomes, as reported in other studies [56–58]. The mechanisms behind the adverse prognosis associated with the male sex are not fully understood, but several hypotheses have been formulated. An explanation could lie in the impact of sex on the pharmacokinetics of rituximab, highlighted in the RICOVER trial [59]. This study demonstrated that rituximab clearance was lower, and the serum elimination half-life was longer in women than in men. Another possible explanation is the contribution of gender-associated gene polymorphisms [60].

Sarcopenia appeared to be related to several factors that may be involved in the prognosis, namely, extranodal involvement, prealbumin, alpha-1 acid glycoprotein, CRP, BMI, and NIS. Several of these factors are nutritional and inflammatory biomarkers, indicating that sarcopenia reflects a complex biological process strongly related to inflammation and nutrition. Interestingly, among nutritional parameters, sarcopenia was associated with prealbumin rather than albumin, suggesting a somewhat acute process. Sarcopenia was not associated with the various geriatric scales, but a lack of power regarding these secondary endpoints is possible. We did not find any evidence of an association between sarcopenia and toxicity or treatment disruption. Sarcopenia measured by L3-SMI has been inconsistently associated with treatment toxicity in DLBCL. Several studies showed an association [33, 34], while others showed a contribution of low muscle density rather than low muscle mass [39, 61].

In contrast with the absence of evidence of an effect of sarcopenia on prognosis, NIS, which appears strongly associated with sarcopenia, emerged as an independent prognostic factor. NIS is a well-known prognostic index that has shown prognostic value in various solid neoplasms [25, 62] and in multiple myeloma [63]. A simpler score based on inflammation and nutrition, which includes CRP and albumin, the GPS, has shown prognostic value in patients with DLBCL [22, 64, 65] superior to other inflammation-based prognostic scores [65]. Interestingly, while GPS was strongly predictive in univariate analysis, NIS rather than GPS was retained as a prognostic factor in the multivariate analysis. This finding suggests that the addition of prealbumin and alpha-1 acid glycoprotein improves upon the prognostic value of albumin and CRP. Alpha-1 acid glycoprotein, an acute-phase protein, has shown prognostic value in lymphoma [66, 67] and is related to tumor burden [67]. Prealbumin, as a marker of recent malnutrition, may complement the information provided by albumin, a well-established prognostic factor in older patients with DLBCL [4, 5]. Recently, Merli and colleagues developed an Elderly Prognostic Index (EPI) based on a simplified version of the geriatric assessment, IPI, and hemoglobin level [68]. An analysis of the links between NIS and EPI would deserve particular attention.

The NIS was also associated with more frequent grades 3–5 complications, particularly among patients treated with R-CHOP rather than R-miniCHOP. This is consistent with a study on the impact of NIS on treatment-related toxicity in cancer patients [24], in which Alexandre et al. showed that alterations of NIS are associated with an increased risk of hematological toxicity, probably due to increased exposure to anti-cancer agent therapy.

Surprisingly, we found a more favorable prognosis in patients with visceral adipopenia for both PFS and OS. This result contradicts previous reports of adverse outcomes in adipopenic patients [38]. An explanation could be a difference in the proportion of overweight and obese patients between the two studies. Adipopenic patients are less likely to be overweight or obese. The prognosis associated with BMI remains controversial in patients with DLBCL [69], with some studies finding better outcomes in overweight patients [70] while obesity was associated with adverse outcomes in other studies [71]. In our study, there was no significant effect of obesity with few patients involved (*n* = 15), but the estimated HRs for obesity of 1.59 for PFS and 1.60 for OS were compatible with an adverse prognosis that may counterbalance the effect of adipopenia in slimmer patients. In the RICOVER study [59], patients with higher weights benefited less from immunochemotherapy than patients with lower weights. Therefore, it is possible that adipopenic patients in our study experienced more favorable outcomes because of better drug exposure [69]. Another explanation for the favorable prognosis associated with visceral adipopenia may be a visceral fat accumulation but subcutaneous fat depletion in higher risk diseases, as is suggested by the association between visceral adipopenia and low R-IPI shown by Lucijanic et al. [72].

## Conclusion

We did not demonstrate any prognostic impact of sarcopenia in older patients with DLBCL. However, sarcopenia was associated with several markers of nutrition, inflammation, and tumor burden. Additionally, we showed that nutritional and inflammatory status, easily calculated from inflammation and nutritional biomarkers, is an independent factor for both prognosis and treatment toxicity in this population. Nutritional parameter improvement appears to be a crucial goal of personalized medicine. The next step would be an interventional study to assess the efficacy of early nutritional support.

### Supplementary information

Supplementary file 1 Supplementary Figure 1- Progression-free survival and overall survival according to the type of chemotherapy in non-sarcopenic patients (PFS (A) and OS (B)) and sarcopenic patients (PFS (C) and OS (D)) (PDF 193 kb)

Supplementary file 2 Supplementary Figure 2 Progression-free survival and overall survival according to the type of chemotherapy in patients with NIS > 1 (PFS (A) and OS (B)) (PDF 159 kb)

Supplementary file 3 Supplementary Table 1- Number of cycles administered and toxicity during the first cycle of chemotherapy according to the type of chemotherapy (PDF 128 kb)

Supplementary file 4 Supplementary Table 2- 2-year PFS and OS according to patients characteristics (DOCX 23 kb)

Supplementary file 5 Supplementary Table 3- Multivariate analysis for progression-free survival and overall survival with multiple imputation for missing values (DOCX 23 kb)

## Data Availability

The datasets used and/or analyzed during the current study are available from the corresponding author on request.

## Abbreviations

DLBCL: diffuse large B-cell lymphoma
R-CHOP: rituximab, cyclophosphamide, doxorubicin, vincristine, and prednisone
IADL: instrumental activity of daily living
CIRS-G: Cumulative Illness Rating Score for Geriatrics
NIS: nutritional and inflammatory status
CRP: C-reactive protein
OS: overall survival
PFS: progression-free survival
CT scan: computed tomography scan
BMI: body mass index
MNA: Mini Nutritional Assessment
PNI: Prognostic Nutritional Index
GNRI: Geriatric Nutritional Risk Index
GPS: Glasgow Prognostic Score
L3-SMI: lumbar L3 skeletal muscle index
L3-VAI: lumbar L3 visceral adipose tissue index
L3-SAI: lumbar L3 visceral subcutaneous tissue index
IPI: International Prognostic Index
ECOG-PS: Eastern Cooperative Oncology Group performance status
LDH: lactate dehydrogenase
HR: hazard ratio
CI: confidence interval
EPI: Elderly Prognostic Index
