# Correction to: Prognostic relevance of sarcopenia, geriatric, and nutritional assessments in older patients with diffuse large B‑cell lymphoma: results of a multicentric prospective cohort study

**DOI:** 10.1007/s00277-023-05497-8

**Published:** 2023-10-26

**Authors:** Juliette Pénichoux, Hélène Lanic, Caroline Thill, Anne-Lise Ménard, Vincent Camus, Aspasia Stamatoullas, Emilie Lemasle, Stéphane Leprêtre, Pascal Lenain, Nathalie Contentin, Jérôme Kraut-Tauzia, Christophe Fruchart, Leïla Kammoun, Gandhi Damaj, Agathe Farge, Caroline Delette, Romain Modzelewski, Sandrine Vaudaux, Louis-Ferdinand Pépin, Hervé Tilly, Fabrice Jardin

**Affiliations:** 1 Department of Clinical Hematology, Centre Henri Becquerel, 1 Rue d’Amiens, 76038 Rouen, France; 2 Department of Statistics, Rouen University Hospital, Rouen, France; 3 INSERM U1245 Unit, Team “Genetic and Biomarkers in Lymphoma and Solid Tumors”, Rouen University, Centre Henri Becquerel, Rouen, France; 4 Wilson Imaging Center, Strasbourg, France; 5 Department of Clinical Hematology, CHD, Dunkirk, France; 6 Department of Oncology‑Hematology, Eure-Seine Hospital Center, Evreux, France; 7 Institute of Hematology, Caen University Hospital, Caen, France; 8 Department of Clinical Hematology, Amiens University Hospital, Amiens, France; 9 Department of Nuclear Medicine, Centre Henri Becquerel, Rouen, France; 10 Clinical Research Unit, Henri Becquerel Cancer Center, Rouen, France

**Correction to** **:** **Annals of Hematology**

**https://doi.org/10.1007/s00277-023-05200-x**

In the published paper, the list of authors is incorrect, as all the authors last names and first names were reversed.

The correct list of authors (first names then last names) is: Juliette Pénichoux, Hélène Lanic, Caroline Thill, Anne-Lise Ménard, Vincent Camus, Aspasia Stamatoullas, Emilie Lemasle, Stéphane Leprêtre, Pascal Lenain, Nathalie Contentin, Jérôme Kraut-Tauzia, Christophe Fruchart, Leïla Kammoun, Gandhi Damaj, Agathe Farge, Caroline Delette, Romain Modzelewski, Sandrine Vaudaux, Louis-Ferdinand Pépin, Hervé Tilly, Fabrice Jardin.

The original article has been corrected.

