# Supplementary material for: Prognostic relevance of sarcopenia, geriatric, and nutritional assessments in older patients with diffuse large B-cell lymphoma: results of a multicentric prospective cohort study
Source: Ann Hematol. 2023 Apr 14;102(7):1811–23. doi: 10.1007/s00277-023-05200-x (PMC10260702; doi:10.1007/s00277-023-05200-x)
Supplement: Supplementary file 1 — Supplementary Figure 1- Progression-free survival and overall survival according to the type of chemotherapy in non-sarcopenic patients (PFS (A) and OS (B)) and sarcopenic patients (PFS (C) and OS (D)) (PDF 193 kb) [file 277_2023_5200_MOESM1_ESM.pdf]

Supplementary Figure 1- Progression-free survival and overall survival according to the type of chemotherapy in non-sarcopenic patients (PFS (A) and OS (B)) and sarcopenic patients (PFS (C) and OS (D))

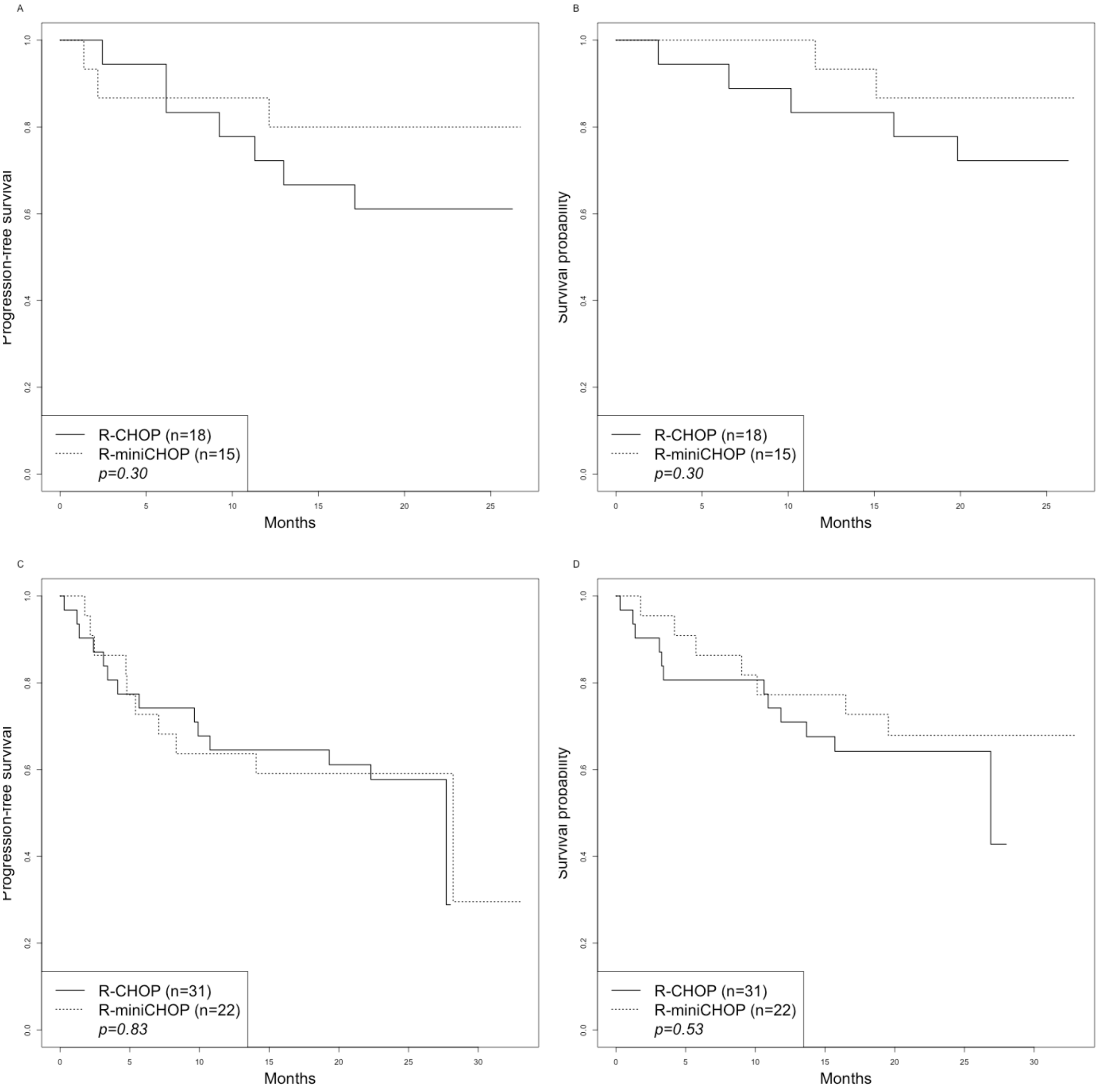

Prognostic relevance of sarcopenia, geriatric, and nutritional assessments in older patients with diffuse large B-cell lymphoma: results of a multicentric prospective cohort study. *Annals of Hematology*

Pénichoux Juliette, Lanic Hélène, Thill Caroline, Ménard Anne-Lise, Camus Vincent, Stamatoullas Aspasia, Lemasle Emilie, Leprêtre Stéphane, Lenain Pascal, Contentin Nathalie, Kraut-Tauzia Jerôme, Fruchart Christophe, Kammoun Leila, Damaj Gandhi, Farge Agathe, Delette Caroline, Modzelewski Romain, Vaudaux Sandrine, Pépin Louis-Ferdinand, Tilly Hervé, Jardin Fabrice

Department of Clinical Hematology, Centre Henri Becquerel, Rouen, France
juliette.penichoux@chb.unicancer.fr
