# Supplementary material for: Prognostic relevance of sarcopenia, geriatric, and nutritional assessments in older patients with diffuse large B-cell lymphoma: results of a multicentric prospective cohort study
Source: Ann Hematol. 2023 Apr 14;102(7):1811–23. doi: 10.1007/s00277-023-05200-x (PMC10260702; doi:10.1007/s00277-023-05200-x)
Supplement: Supplementary file 2 — Supplementary Figure 2 Progression-free survival and overall survival according to the type of chemotherapy in patients with NIS > 1 (PFS (A) and OS (B)) (PDF 159 kb) [file 277_2023_5200_MOESM2_ESM.pdf]

Supplementary Figure 2- Progression-free survival and overall survival according to the type of chemotherapy in patients with NIS > 1 (PFS (A) and OS (B))

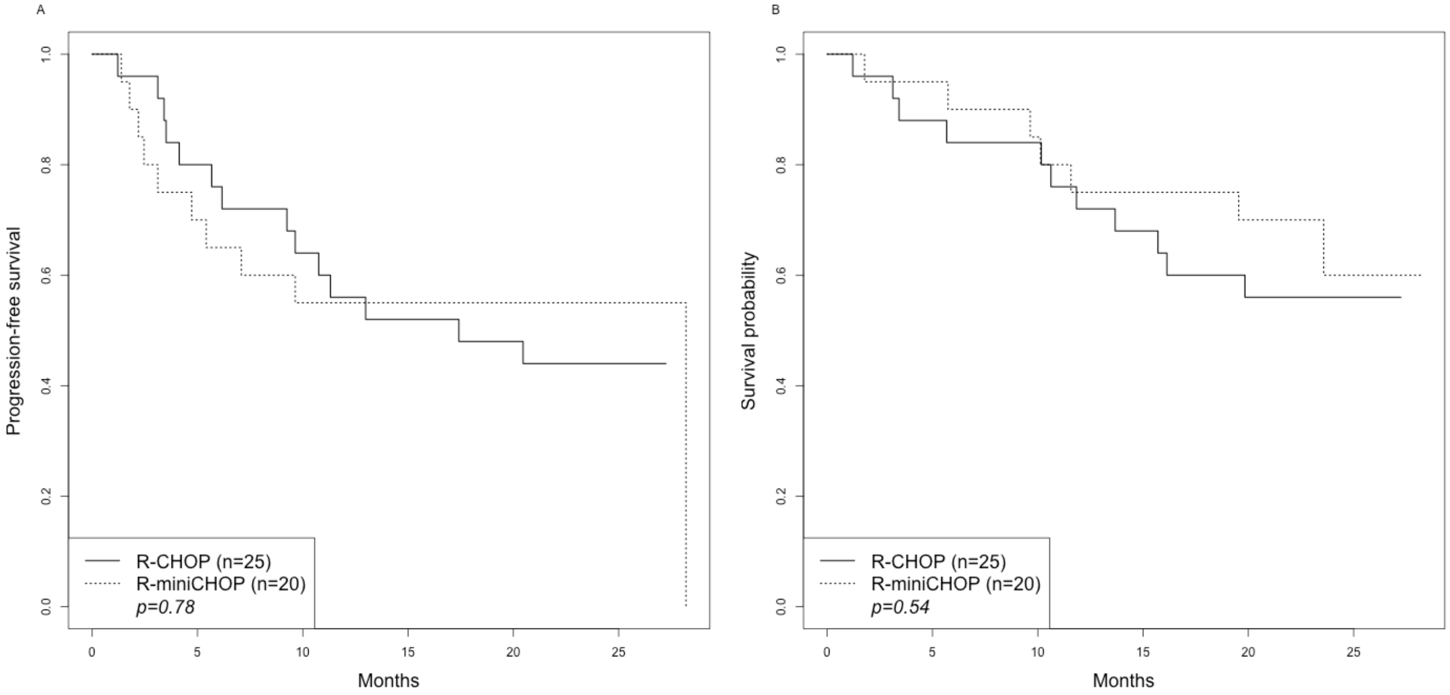

Too few events were reported in patients with NIS < 1 to compare R-CHOP vs. R-miniCHOP in this subgroup

Prognostic relevance of sarcopenia, geriatric, and nutritional assessments in older patients with diffuse large B-cell lymphoma: results of a multicentric prospective cohort study.
Annals of Hematology

Pénichoux Juliette, Lanic Hélène, Thill Caroline, Ménard Anne-Lise, Camus Vincent, Stamatoullas Aspasia, Lemasle Emilie, Leprêtre Stéphane, Lenain Pascal, Contentin Nathalie, Kraut-Tauzia Jerôme, Fruchart Christophe, Kammoun Leila, Damaj Gandhi, Farge Agathe, Delette Caroline, Modzelewski Romain, Vaudaux Sandrine, Pépin Louis-Ferdinand, Tilly Hervé, Jardin Fabrice

Department of Clinical Hematology, Centre Henri Becquerel, Rouen, France
juliette.penichoux@chb.unicancer.fr
