# Supplementary material for: Prognostic relevance of sarcopenia, geriatric, and nutritional assessments in older patients with diffuse large B-cell lymphoma: results of a multicentric prospective cohort study
Source: Ann Hematol. 2023 Apr 14;102(7):1811–23. doi: 10.1007/s00277-023-05200-x (PMC10260702; doi:10.1007/s00277-023-05200-x)
Supplement: Supplementary file 3 — Supplementary Table 1- Number of cycles administered and toxicity during the first cycle of chemotherapy according to the type of chemotherapy (PDF 128 kb) [file 277_2023_5200_MOESM3_ESM.pdf]

Supplementary Table 1- Number of cycles administered and toxicity during the first cycle of chemotherapy according to the type of chemotherapy

	Total			Non-Sarcopenic			Sarcopenic			NIS < 1			NIS > 1		
	R-CHOP (n=54)	R-miniCHOP (n=40)	p	R-CHOP (n=31)	R-miniCHOP (n=22)	p	R-CHOP (n=18)	R-miniCHOP (n=15)	p	R-CHOP (n=16)	R-miniCHOP (n=11)	P	R-CHOP (n=25)	R-miniCHOP (n=20)	P
Number of cycles administered			0.61			1			0.32						1
<6	13 (24%)	7 (18%)		3 (16%)	3 (20%)		9 (29%)	3 (14%)		0	0		6 (24%)	5 (25%)	
6-8	41 (76%)	33 (82%)		15 (83%)	12 (80%)		22 (71%)	19 (86%)		16 (100%)	11 (100%)		19 (76%)	15 (75%)	
Adverse events grade 3-5 during the first cycle of treatment			**0.01**			0.23			0.22			0.41			**0.01**
0	42 (78%)	38 (95%)		15 (83%)	14 (93%)		24 (77%)	21 (95%)		16 (100%)	10 (91%)		18 (72%)	20 (100%)	
1	11 (20%)	1 (3%)		3 (17%)	0		6 (19%)	1 (5%)		0	0		7 (28%)	0	
2	1 (2%)	1 (3%)		0	1 (7%)		1 (3%)	0		0	1 (9%)		0	0	

Prognostic relevance of sarcopenia, geriatric, and nutritional assessments in older patients with diffuse large B-cell lymphoma: results of a multicentric prospective cohort study. *Annals of Hematology*

Pénichoux Juliette, Lanic Hélène, Thill Caroline, Ménard Anne-Lise, Camus Vincent, Stamatoullas Aspasia, Lemasle Emilie, Leprêtre Stéphane, Lenain Pascal, Contentin Nathalie, Kraut-Tauzia Jerôme, Fruchart Christophe, Kammoun Leila, Damaj Gandhi, Farge Agathe, Delette Caroline, Modzelewski Romain, Vaudaux Sandrine, Pépin Louis-Ferdinand, Tilly Hervé, Jardin Fabrice

Department of Clinical Hematology, Centre Henri Becquerel, Rouen, France
juliette.penichoux@chb.unicancer.fr
