# Supplementary material for: Prognostic relevance of sarcopenia, geriatric, and nutritional assessments in older patients with diffuse large B-cell lymphoma: results of a multicentric prospective cohort study
Source: Ann Hematol. 2023 Apr 14;102(7):1811–23. doi: 10.1007/s00277-023-05200-x (PMC10260702; doi:10.1007/s00277-023-05200-x)
Supplement: Supplementary file 4 — Supplementary Table 2- 2-year PFS and OS according to patients characteristics (DOCX 23 kb) [file 277_2023_5200_MOESM4_ESM.docx]

|  | 2-year PFS  (% [95%CI]) | |  | 2-year OS  (% [95%CI]) |
| --- | --- | --- | --- | --- |
| Non-sarcopenic  Sarcopenic | | 70% [51%-82%]  58% [36%-66%] | | 79% [66%-94%]  66%[54%‑80%] |
| Gender, female  Gender, male | | 71% [59%-85%]  48% [36%-65%] | | 73% [61%-88%]  64% [51%-79%] |
| Age $\leq$ 80 yr.  Age > 80 yr. | | 63% [52%-76%]  54% [40%-74%] | | 71% [60%-83%]  63% [46%-85%] |
| Stage I-II  Stage III-IV | | 76% [62%-93%]  52% [41%-66%] | | 83% [71%-98%]  62% [51%-75%] |
| 0-1 extranodal site  > 1 extranodal site | | 66% [55%-80%]  48% [34%-68%] | | 75% [63%-88%]  57% [43%-76%] |
| No B-symptoms  B-symptoms | | 67% [57%-80%]  43% [28%-65%] | | 74% [63%-86%]  56% [41%-77%] |
| No bulky disease  Bulky disease (>10 cm) | | 70% [59%-82%]  42% [28%-62%] | | 75% [64%-88%]  57% [43%-76%] |
| ECOG-PS< 2  ECOG-PS≥ 2 | | 64% [53%-78%]  53% [39%-72%] | | 76% [66%-88%]  57% [42%-76%] |
| IPI < 3  IPI ≥ 3 | | 67% [56%-81%]  47% [33%-66%] | | 78% [68%-90%]  54% [40%-73%] |
| BMI < 25 | | 62% [49%-77%] | | 68% [56%-83%] |
| Overweight (BMI [25 ; 30]) | | 62% [47%-81%] | | 78% [65%-94%] |
| Obese (BMI > 30) | | 47% [27%-80%] | | 50% [29%-86%] |
| No visceral adipopenia*  Visceral adipopenia * | | 52% [40%-68%]  76% [64%-91%] | | 65% [52%-80%]  81% [70%-95%] |
| No subcutaneous adipopenia*  Subcutaneous adipopenia* | | 60% [47%-75%]  69% [54%-87%] | | 70% [58%-84%]  74% [60-91] |
| No lymphopenia  Lymphopenia | | 71% [60%-85%]  47% [34%-64%] | | 84% [74%-95%]  50% [36%-70%] |
| No hypoalbuminemia  Hypoalbuminemia | | 94% [83%-100%]  53% [43%-66%] | | *No event*  62% [52%-75%] |
| NIS $\leq$ 1  NIS > 1 | | 88% [76%-100%]  49% [36%-66%] | | *No event*  58% [44%-76%] |
| LDH$\leq$UNL  LDH>UNL | | 78% [64%-95%]  52% [41%-66%] | | 86% [73%-100%]  64% [53%-77%] |
| Timed Up and Go test <20s | | 66% [54%-81%] | | 74% [63%-87%] |
| Timed Up and Go test >20s or impossible | | 47% [29%-76%] | | 58% [39%-85%] |
| Hand grip test (left) > median  Hand grip test (left) $\leq$ median | | 57% [44%-73%]  63% [51%-79%] | | 72% [60%-88%]  66% [54%-81%] |
| Hand grip test (left) > median  Hand grip test (right)$\leq$ median | | 57% [44%-73%]  64% [51%-79%] | | 68% [56%-83%]  70% [57%-86%] |
| G8≥14  G8 < 14 | | 60% [44%-83%]  59% [48%-72%] | | 68% [52%-89%]  68% [57%-81%] |
| MNA | |  | |  |
| <17 | | 75% [43%-100%] | | 75% [43%-100%] |
| 17-24 | | 62% [49%-78%] | | 64% [51%-80%] |
| >24 | | 56% [41%-76%] | | 81% [69%-96%] |
| IADL score (/4) ≥4  IADL score (/4) <4 | | 63% [52%-75%]  50% [33%-76%] | | 75% [66%-86%]  41% [21%-81%] |
| CIRS-G score $\leq$7  CIRS-G score >7 | | 60% [50%-73%]  54% [37%-78%] | | 71% [61%-82%]  60% [42%-85%] |
| PNI ≥ 45  PNI < 45 | | 72% [56%-92%]  55% [44%-69%] | | 88% [77%-100%]  61% [50%-75%] |
| GNRI, categories  No risk  Low risk  Moderate risk  High risk | | 75% [59%-94%]  61% [44%-83%]  72% [56%-92%]  13% [4%-48%] | | 83% [70%-100%]  70% [51%-97%]  76% [61%-95%]  27% [11%-62%] |
| GPS | |  | |  |
| 0 | | 84% [71%-100%] | | 96% [89%-100%] |
| 1 | | 56% [40%-78%] | | 67% [51%-87%] |
| 2 | | 43% [29%-65%] | | 48% [33%-70%] |

Supplementary Table 2- 2-year PFS and OS according to patients characteristics

PFS, progression-free survival; OS, overall survival; ECOG-PS, Eastern Cooperative Oncology Group performance status; IPI, International Prognostic Index; BMI, body mass index; NIS, nutritional and inflammatory status; LDH lactate dehydrogenase; ULN, upper normal limit; MNA mini nutritional assessment; IADL, Instrumental Activities of Daily Living; CIRS-G, Cumulative Illness Rating Scale-Geriatric; PNI, Prognostic Nutritional Index; GNRI, Geriatric Nutritional Risk Index; GPS, Glasgow Prognostic Score

* Visceral adipopenia: male L3-VAI < 50.4 cm^2^/m^2^, female L3-VAI < 43.5 cm^2^/m^2^

Subcutaneous adipopenia: male L3-SAI < 47.4 cm^2^/m^2^, female L3-SAI < 76.3 cm^2^/m^2^
