# Supplementary material for: Prognostic relevance of sarcopenia, geriatric, and nutritional assessments in older patients with diffuse large B-cell lymphoma: results of a multicentric prospective cohort study
Source: Ann Hematol. 2023 Apr 14;102(7):1811–23. doi: 10.1007/s00277-023-05200-x (PMC10260702; doi:10.1007/s00277-023-05200-x)
Supplement: Supplementary file 5 — Supplementary Table 3- Multivariate analysis for progression-free survival and overall survival with multiple imputation for missing values (DOCX 23 kb) [file 277_2023_5200_MOESM5_ESM.docx]

|  |  | PFS | | | | |  | OS | | | | |
| --- | --- | --- | --- | --- | --- | --- | --- | --- | --- | --- | --- | --- |
|  |  | Univariate | |  | Multivariate | |  | Univariate | |  | Multivariate | |
|  |  | HR [95%CI] | p |  | HR [95%CI] | p |  | HR [95%CI] | p |  | HR [95%CI] | p |
| Sarcopenia | | 1.63 [0.78 – 3.43] | 0.18 |  | 0.81 [0.36-1.86] | 0.61 |  | 1.91 [0.77-4.68] | 0.15 |  | 0.97 [0.34-2.72] | 0.95 |
| Gender, female | | 0.51 [0.27 – 0.98] | **0.04** |  | 0.44 [0.21-0.93] | **0.03** |  | 0.59 [0.28-1.23] | 0.15 |  | 0.60 [0.25-1.43] | 0.23 |
| Age > 80 yr. | | 1.41 [0.75-2.66] | 0.29 |  | - |  |  | 1.28 [0.62-2.67] | 0.50 |  | - |  |
| Stage III-IV | | 2.69 [1.19 – 6.10] | **0.01** |  | - |  |  | 2.02 [0.82-4.94] | 0.10 |  | - |  |
| > 1 extranodal site | | 1.96 [1.05-3.65] | **0.03** |  | - |  |  | 2.32 [1.13-4.76] | **0.02** |  | - |  |
| B-symptoms | | 2.14 [1.14-3.99] | **0.02** |  | - |  |  | 1.92 [0.93-3.97] | 0.08 |  | - |  |
| Bulky disease (>10 cm) | | 2.03 [1.09-3.78] | **0.03** |  | 2.14 [1.02-4.47] | **0.04** |  | 1.96 [0.96-4.02] | 0.06 |  | 1.71 [0.73-4.04] | 0.20 |
| ECOG-PS≥ 2 | | 1.43 [0.76-2.70] | 0.27 |  | - |  |  | 1.84 [0.90-3.77] | 0.10 |  | - |  |
| IPI ≥ 3 | | 2.15 [1.12-4.09] | **0.02** |  | 0.92 [0.44-1.92] | 0.81 |  | 2.26 [1.06-4.84] | **0.04** |  | 1.05 [0.44-2.47] | 0.91 |
| BMI (kg/m2) | |  |  |  | - |  |  |  |  |  | - |  |
| < 25 | | 1 |  |  | - |  |  | 1 |  |  | - |  |
| Overweight ([25 ; 30]) | | 0.94 [0.45-1.97] | 0.88 |  | - |  |  | 0.75 [0.30-1.85] | 0.51 |  | - |  |
| Obese (> 30) | | 1.51 [0.63-3.59] | 0.34 |  | - |  |  | 1.53 [0.59-3.95] | 0.37 |  | - |  |
| Low L3-VAI* | | 0.49 [0.23-1.04] | **0.06** |  | 0.37 [0.97-6.81] | 0.06 |  | 0.60 [0.25-1.46] | 0.25 |  | 0.37 [0.12-1.14] | 0.08 |
| Low L3-SAI * | | 0.67 [0.30-1.49] | 0.32 |  | - | - |  | 0.65 [0.26-1.62] | 0.33 |  | - | - |
| Lymphopenia | | 2.58 [1.34-4.99] | **0.005** |  | 2.07 [0.94-4.60] | 0.07 |  | 3.26 [1.49-7.16] | **0.003** |  | 2.55 [1.00-6.54] | **0.05** |
| Hypoalbuminemia | | 12.18 [1.56 – 95.74] | **0.02** |  | 5.99 [0.65-55.65] | 0.11 |  | 8.01 [0.99-64.84] | **0.05** |  | 2.28 [0.22-23.63] | 0.47 |
| NIS > 1 | | 5.56 [2.09-14.78] | **0.001** |  | 3.50 [1.15-10.59] | **0.03** |  | 9.57 [2.12-43.1] | **0.01** |  | 5.29 [0.99 -28.10] | **0.05** |
| LDH>UNL | | 2.45 [1.05-5.69] | **0.04** |  | - |  |  | 2.40 [0.88-6.55] | **0.09** |  | - |  |
| Timed Up and Go test >20s | | 1.68 [0.82-3.45] | 0.15 |  | - |  |  | 1.84 [0.79-4.29] | 0.15 |  | - |  |
| Hand grip test (left) | | 1.00 [0.98-1.02] | 0.80 |  | - |  |  | 1.01 [0.99-1.03] | 0.54 |  | - |  |
| Hand grip test (right) | | 1.00 [0.98-1.02] | 0.77 |  | - |  |  | 1.00 [0.98-1.03] | 0.76 |  | - |  |
| G8 < 14 | | 0.89 [0.43-1.83] | 0.74 |  | - |  |  | 0.95 [0.41-2.23] | 0.91 |  | - |  |
| MNA | |  |  |  | - |  |  |  |  |  | - |  |
| <17 | | 1.16 [0.21-6.52] | 0.63 |  | - |  |  | 2.02 [0.30-13.53] | 0.44 |  | - |  |
| 17-24 | | 1.09 [0.52-2.26] | 0.91 |  | - |  |  | 1.77 [0.70-4.45] | 0.21 |  | - |  |
| >24 | | 1 |  |  | - |  |  | 1 |  |  | - |  |
| IADL score (/4) <4 | | 1.50 [0.73-3.10] | 0.27 |  | - |  |  | 2.33 [1.06-5.12] | **0.04** |  | - |  |
| CIRS-G score >7 | | 1.38 [0.67-2.87] | 0.37 |  | - |  |  | 1.31 [0.58-2.99] | 0.50 |  | - |  |
| PNI < 45 | | 2.32 [0.99-5.42] | **0.05** |  | - |  |  | 3.10 [1.03-9.34] | **0.05** |  | - |  |
| GNRI, categories | | 1.87 [1.32-2.66] | **0.001** |  | - |  |  | 1.95 [1.30-2.92] | **0.002** |  | - |  |
| GPS | |  |  |  | - |  |  |  |  |  | - |  |
| 0 | | 1 |  |  |  |  |  | 1 |  |  |  |  |
| 1 | | 4.04 [1.25-13.06] | **0.02** |  |  |  |  | 4.90 [0.97-24.60] | **0.05** |  |  |  |
| 2 | | 6.79 [2.15-21.41] | **0.002** |  |  |  |  | 10.04 [2.05-49.09] | **0.01** |  |  |  |

Supplementary Table 3- Multivariate analysis for progression-free survival and overall survival with multiple imputation for missing values

PFS, progression-free survival; OS, overall survival; HR, hazard ratio; CI, confidence interval; ECOG-PS, Eastern Cooperative Oncology Group performance status; IPI, International Prognostic Index; BMI, body mass index; L3-VAI, lumbar L3 visceral adipose tissue index; L3-SAI, lumbar L3 subcutaneous adipose tissue index; NIS, nutritional and inflammatory status; LDH lactate dehydrogenase; ULN, upper normal limit; MNA mini nutritional assessment; IADL, Instrumental Activities of Daily Living; CIRS-G, Cumulative Illness Rating Scale-Geriatric; PNI, Prognostic Nutritional Index; GNRI, Geriatric Nutritional Risk Index; GPS, Glasgow Prognostic Score

* Low L3-SAI: male L3-SAI < 47.4 cm^2^/m^2^, female L3-SAI < 76.3 cm^2^/m^2^

Low L3-VAI: male L3-VAI < 50.4 cm^2^/m^2^, female L3-VAI < 43.5 cm^2^/m^2^

Prognostic relevance of sarcopenia, geriatric, and nutritional assessments in older patients with diffuse large B-cell lymphoma: results of a multicentric prospective cohort study. *Annals of Hematology*

Pénichoux Juliette, Lanic Hélène, Thill Caroline, Ménard Anne-Lise, Camus Vincent, Stamatoullas Aspasia, Lemasle Emilie, Leprêtre Stéphane, Lenain Pascal, Contentin Nathalie, Kraut-Tauzia Jerôme, Fruchart Christophe, Kammoun Leila, Damaj Gandhi, Farge Agathe, Delette Caroline, Modzelewski Romain, Vaudaux Sandrine, Pépin Louis-Ferdinand, Tilly Hervé, Jardin Fabrice

Department of Clinical Hematology, Centre Henri Becquerel, Rouen, France

juliette.penichoux@chb.unicancer.fr
